# Biomarcadores de estrés oxidativo e inflamación en lesión renal aguda postcontraste yodado

**DOI:** 10.23938/ASSN.1081

**Published:** 2024-08-05

**Authors:** Elisabeth Stoll, Pablo Monedero, Paloma L. Martín-Moreno, Nuria García-Fernández

**Affiliations:** 1 Clínica Universidad de Navarra Departamento de Anestesia y Cuidados Intensivos Pamplona España; 2 Asklepios Kinderklinik Sankt Augustin Departamento de Anestesia Pediátrica Sankt Augustin Alemania; 3 Clínica Universidad de Navarra Departamento de Nefrología Pamplona España; 4 Instituto de Investigación Sanitaria de Navarra (IdiSNA) Pamplona España; 5 RICORS2040 (Kidney Disease) Pamplona España

**Keywords:** Lesión renal aguda, Biomarcadores, Contraste yodado, Tomografía computarizada, Iohexol, Acute kidney injury, Biomarkers, Iodinated contrast, Computed tomography, Iohexol

## Abstract

**Fundamento:**

La lesión renal aguda postcontraste yodado (LRAPCY) es una causa frecuente de insuficiencia renal, especialmente en pacientes con factores de riesgo. Este estudio analiza diferentes biomarcadores renales en pacientes sometidos a tomografía computarizada con contraste yodado para identificar los mecanismos moleculares y celulares implicados en la patogénesis de la LRAPCY.

**Metodología:**

Estudio prospectivo de pacientes de alto riesgo renal que recibieron contraste yodado (iohexol) para tomografía computarizada. Se analizaron biomarcadores funcionales (creatinina y cistatina C), de inflamación y estrés oxidativo (lipocalina asociada a gelatinasa de neutrófilos [NGAL], interleucina-8 [IL-8], superóxido dismutasa [SOD], F2-isoprostanos y cardiotrofina-1) y de ciclo celular (Nephrocheck®), precontraste y 4, 12, 24 y 48 horas postcontraste, en relación con la LRAPCY.

**Resultados:**

Se observó LRAPCY en el 30,6% de los 62 pacientes incluidos y en el 57,1% de los pacientes con diabetes y disfunción renal. Los factores asociados con LRAPCY fueron: mayor edad media (74,4 *vs* 64,9 años), existencia de disfunción renal previa (60 *vs* 16,7%), y mayor volumen medio ajustado de iohexol (42,9 *vs* 32,1%). Los pacientes con y sin LRAPCY no difirieron respecto a los biomarcadores no-funcionales. Se observó una disminución de la actividad antioxidante de la SOD a las 4 horas y un aumento de IL-8 a las 12 horas post-administración del contraste yodado.

**Conclusiones:**

La administración de iohexol en tomografía computarizada en pacientes con alto riesgo de enfermedad renal resulta en un elevado porcentaje de LRAPCY, atribuible a daño por isquemia/reperfusión y/o toxicidad directa del contraste yodado.

## Abstract (en)

**Background:**

Iodinated contrast-induced acute kidney injury (CI-AKI) is a common cause of renal failure, especially in patients with risk factors. This study analyses different renal biomarkers in patients undergoing computed tomography scans with iodinated contrast to identify the molecular and cellular mechanisms involved in the pathogenesis of CI-AKI.

**Methodology:**

Prospective study that included patients with high risk of renal disease who received iodinated contrast (iohexol) for the computed tomography scans. Functional biomarkers (creatinine and cystatin C), inflammatory and oxidative stress markers (neutrophil gelatinase-associated lipocalin [NGAL], interleukin-8 [IL-8], superoxide dismutase [SOD], F2-isoprostanes, and cardio-trophin-1), and cell cycle biomarkers (Nephrocheck®) were analysed before the iodinated contrast and 4, 12, 24, and 48 hours post-contrast, in relation to the incidence of IC-AKI.

**Results:**

IC-AKI was observed in 30.6% of the 62 study participants and in 57.1% of the patients with diabetes and renal dysfunction. Factors associated with IC-AKI were a higher mean age (74.4 *vs* 64.9 years), pre-existing renal dysfunction (60 *vs* 16.7%), and higher adjusted mean volume of iohexol (42.9 *vs* 32.1%). As for non-functional biomarkers. No differences were found between patients with and without CI-AKI. The use of iodinated contrast was associated with a decrease in SOD antioxidant activity at 4 hours and an increase in IL-8 at 12 hours post-administration of the iodinated contrast.

**Conclusions:**

Administration of iohexol in computed tomography scans in patients with high risk of renal disease results in an elevated percentage of CI-AKI, attributable to ischemia/ reperfusion injury and/or direct toxicity of the iodinated contrast.

## INTRODUCCIÓN

La lesión renal aguda postcontraste yodado (LRAPCY) es una causa frecuente de insuficiencia renal, especialmente en pacientes con factores de riesgo como enfermedad renal crónica (ERC) preexistente, edad avanzada y otras comorbilidades que incrementan la incidencia de daño renal, tales como diabetes mellitus, hipovolemia, uso de nefrotóxicos, hipotensión, albuminuria e insuficiencia cardíaca^1^.

La LRAPCY es un término amplio que se refiere al daño renal que ocurre poco después de la administración de contraste yodado, independientemente de si es causado directamente por el material de contraste^1^. Este síndrome es complejo y multifactorial, caracterizado por un espectro de agresiones patobiológicas complicadas por la multimorbilidad del paciente^2^. Los contrastes yodados intravasculares se han considerado nefrotóxicos a partir de experimentos con animales, estudios no controlados en humanos y observaciones anecdóticas^1^. El iohexol es un contraste yodado de uso frecuente en nuestro medio que, a pesar de ser no-iónico y de baja osmolaridad, ha inducido nefrotoxicidad *in vivo* (en modelos animales) debido a apoptosis, disminución de las enzimas antioxidantes con activación del inflamasoma NLRP3, daño mitocondrial y mitofagia^3^.

Los mecanismos propuestos de patogénesis de la LRAPCY incluyen principalmente cambios hemodinámicos en el riñón, como la vasoconstricción y la disminución del flujo sanguíneo renal, así como la lesión tubular directa por yodo^4^. Los contrastes yodados provocan diuresis osmótica, un aumento de la presión tubular y una disminución del flujo tubular y sanguíneo, lo cual conduce a un aumento de la demanda tubular de oxígeno y una disminución del suministro de sangre renal. Esto, unido a la vasoconstricción directa de los vasos rectos a través de disfunción endotelial, empeora la hipoxia medular del riñón. Este desajuste entre las demandas metabólicas del túbulo y el suministro de sangre medular del riñón conduce al estrés oxidativo con la producción de especies reactivas de oxígeno (ROS). El daño isquémico y citotóxico de las células tubulares induce nuevamente retroalimentación túbulo-glomerular, intensificando la vasoconstricción de la arteriola aferente y produciendo disminuciones adicionales en el flujo sanguíneo renal y la TFG^5^^-^^8^. LRAPCY se caracteriza por una recuperación relativamente rápida de la función renal en pocos días, lo cual puede explicarse por una lesión menos grave o por la presencia de cambios funcionales en lugar de necrosis en las células epiteliales de los túbulos renales.

Dado que la patogénesis de la LRAPCY es multifactorial y no completamente aclarada, el objetivo de nuestro estudio es caracterizar la fisiopatología de la LRAPCY e identificar los mecanismos moleculares y celulares implicados en su patogénesis mediante el análisis de biomarcadores relacionados con la función, la inflamación, el estrés oxidativo y el ciclo celular en pacientes con alto riesgo renal que iban a recibir contraste yodado para la realización de una tomografía computarizada.

## MATERIAL Y MÉTODOS

Estudio observacional prospectivo realizado en la Clínica Universidad de Navarra (Pamplona, España) entre agosto de 2008 y mayo de 2013 con pacientes hospitalizados con factores de riesgo para el desarrollo de daño renal agudo (LRA) y que recibieron contraste yodado previamente a la realización de una tomografia computarizada (TC).

Los criterios de inclusión fueron: ser mayor de edad (≥18 años), hospitalización mínima de 60 horas (12 horas antes de la TC y 48 horas después), someterse a una TC con administración intravenosa de contraste yodado Omnipaque® (iohexol: medio de contraste no iónico de baja osmolaridad 410-780 mOsm/kg H_2_O, 180-350 mg/mL de contenido de yodo, y 2,2-10,6 mPa*s de viscosidad a 37 ºC) y presentar al menos un factor de riesgo de LRA: tasa de filtración glomerular estimada (FGe) mediante MDRD-4 (fórmula recomendada en el momento de confección del protocolo) <60 mL/min/1,73 m^2^; diabetes mellitus; administración de contraste yodado en la semana previa; y/o uso de fármacos nefrotóxicos, como antiinflamatorios no esteroides (AINES), antibióticos nefrotóxicos, quimioterapia nefrotóxica e inmunosupresores, o colonoscopia en las 48 horas previas a la inyección de contraste,

Se excluyeron pacientes con antecedentes de reacciones graves a los medios de contraste, en hemodiálisis, con insuficiencia cardíaca clase III-IV de la *New York Heart Association* (NYHA), exacerbaciones agudas de enfermedad pulmonar obstructiva crónica e hipertensión arterial resistente definida como cifras mayores de 150/90 mm Hg a pesar del tratamiento antihipertensivo.

El comité de ética del hospital aprobó el protocolo del estudio (NIC2117; EudraCT: 2008-000621-19) y todos los pacientes incluidos dieron su consentimiento informado por escrito.

Todos los pacientes recibieron hidratación intravenosa u oral, según prescripción médica, antes de la administración del contraste.

Se recogieron las siguientes variables de cada paciente:

- características demográficas: edad (años), sexo (mujer, varón);
- características clínicas: índice de masa corporal (IMC, en kg/m^2^), presión arterial sistólica (PAS, mm Hg), diastólica (PAD, mm Hg) y media (PAM, mm Hg);
- factores de riesgo de LRA/comorbilidades: diabetes mellitus (DM), hipertensión arterial (HTA), ERC (FGe_(MDRD)_ <60 mL/min/1,73 m^2^, categorizada en estadios KDIGO G3A, G3B y G4), uso de antiinflamatorios no-esteroides (AINES), uso de inhibidores de la enzima de conversión de la angiotensina o de antagonistas de los receptores de la angiotensina II (IECA), ausencia de ingesta o colonoscopia dentro de las 48 horas previas (deshidratación en ayunas), anemia (hemoglobina <14 g/dL en varones y <12 g/dL en mujeres);
- factores protectores: hidratación mediante fluidoterapia previa, y administración de N-acetilcisteína intravenosa, ambos previamente al uso de iohexol;
- variables en relación con la administración de contraste: dosis de contraste aceptable máxima (MACD: *maximal acceptable contrast dose*), calculada como 5 mL*peso corporal (kg)/creatinina basal en sangre (mg/dL), con una dosis máxima de 300 mL^9^, volumen de iohexol recibido normalizado segun el volumen de la MACD (mL iohexol/mL MACD).

Todos los parámetros bioquímicos generales se determinaron por métodos estándar en el laboratorio de bioquímica de nuestro hospital.

**Figura 1:**
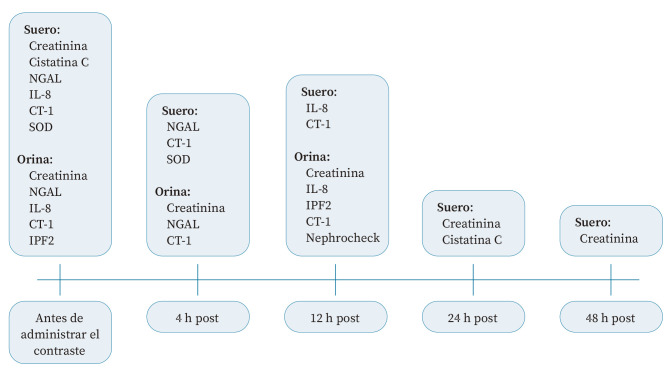
Cronograma de determinaciones analíticas.

Las determinaciones de biomarcadores se realizaron según el cronograma mostrado en la Figura 1. Se extrajeron muestras de sangre antes de la administración del contraste, así como 4, 12, 24 y 48 horas después. Se recogió la orina desde 12 horas antes y durante 12 horas después de la administración de contraste, con muestreo a las 4 y 12 horas; todos los parámetros urinarios se normalizaron mediante la creatinina urinaria.

*Biomarcadores de función renal*:

- Diuresis (mL/h)
- Creatinina (mg/dL): la concentración plasmática (rango normal: 0,7-1,3 mg/dL en varones y 0,6-1,1 mg/dL en mujeres) y urinaria se midió en el analizador Synchron LX20 (Beckman-Coulter; Beckman, Fullerton, California, EEUU) mediante la reacción de Jaffe;
- Cistatina C (mg/L, basal, 24h): su concentración sérica (rango normal: 0,62-1,11 mg/L) se midió mediante nefelometría utilizando una plataforma de laboratorio clínico estandarizada (BN Siemens, Marburg Alemania);
- FGe (ml/min; MDRD): tasa de filtración glomerular estimada mediante MDRD-4;
- FGe (mL/min; CKD-EPI creat-cist): tasa de filtración glomerular estimada mediante *Chronic Kidney Disease Epidemiology Collaboration* creatinina-cistatina.

*Biomarcadores relacionados con la inflamación*:

- Lipocalina asociada a la gelatinasa de neutrófilos (NGAL, ng/mL); su concentración en suero (valor normal ≤400 ng/mL) y en orina (valor normal ≤150ng/mL) se analizó con kits ELISA disponibles comercialmente (BioPorto Diagnostics, Hellerup, Dinamarca); se diluyó 1/10 en plasma y 1/50 en orina;
- Interleucina-8 humana (IL-8, pg/mL); su concentración en suero (valor normal ≤65 pg/mL) y orina (valor normal <15 pg/mL) se determinó mediante el inmunoensayo Quantikine® Human CXCL8/IL-8 (R&D Systems, Minnesota, EEUU) de acuerdo con las instrucciones del fabricante;
- Cardiotrofina-1 (CT-1, fmol/mL); su concentración sérica (valor normal ≤40 fmol**/**mL) y urinaria se analizó mediante un ensayo ELISA desarrollado en el Centro de Investigación Médica Aplicada (CIMA) de la Universidad de Navarra (Pamplona, España); se utilizó un anticuerpo monoclonal anti-CT-1 de ratón como anticuerpo de captura y un anticuerpo policlonal biotinilado anti-CT-1 de rata (MAB 438) como anticuerpo de detección (I+D)^10^, a una dilución 1/20 para plasma y 1/10 para orina.

*Biomarcadores relacionados con el estrés oxidativo:*

- Superóxido dismutasa (SOD, U/mL); en plasma se analizó con el ensayo de SOD (Cayman Laboratories, Ann Arbor, Michigan, EE. UU.) que utiliza una sal de tetrazolio para la detección de radicales superóxidos generados por la xantina oxidasa y la hipoxantina. Mide los tres tipos de SOD (Cu/Zn-, Mn- y Fe-SOD). Una unidad de SOD se define como la cantidad de enzima necesaria para exhibir una dismutación del 50% del radical superóxido;
- F2-isoprostanos urinarios (IPF2, pg/mg); en orina (valor normal <3.000 pg/mL) se determinaron mediante el KIT EIA iPF2α-VI (Cayman Laboratories, Ann Arbor, Michigan, EEUU), un método fiable para medir la familia de isoprostanos de clase VI (una de las cuatro clases principales: III, IV, V y VI);
- F2-isoprostanos urinarios ajustados a creatinina en orina (IPF2/creat, pg/mg).

*Biomarcadores del ciclo celular:*

- Test NephroCheck®: mide cuantitativamente en orina las proteínas TIMP-2 (inhibidor tisular de metaloproteinasa 2) e IGFBP-7 (proteína transportadora del factor de crecimiento insulínico 7) en una tira reactiva de membrana que emplea una técnica de inmunoanálisis de fluorescencia tipo sándwich, con un analizador que convierte la señal fluorescente de las dos concentraciones de inmunoanálisis en un único resultado numérico. Un resultado elevado, >0,3 (ng/mL)^2^/1.000, indica un riesgo elevado de LRA.

Todas las muestras se dividieron en alícuotas y se almacenaron a -80 ºC hasta su procesamiento. Todas las mediciones se realizaron por duplicado.

El resultado primario fue la lesión renal aguda poscontraste yodado (LRAPCY), definida según los criterios del grupo de consenso *Kidney Disease: Improving Global Outcomes* (KDIGO) para la lesión renal aguda como un aumento en la creatinina sérica ≥0,3 mg/dL respecto al valor inicial dentro de las 48 horas posteriores a la administración de contraste, o una diuresis <0,5 mL/kg/h durante al menos 6 horas^11^. Evaluamos la asociación entre LRAPCY y los datos demográficos, factores de riesgo y niveles de biomarcadores de función, inflamación, estrés oxidativo y de ciclo celular. Además, se valoró la evolución de los biomarcadores renales tras la administración de contraste^12^.

Se utilizó la prueba de Kolmogorov-Smirnov para comprobar la distribución normal de los datos. Las características demográficas y los valores bioquímicos de los participantes se expresaron como media y desviación estándar (DE), o mediana y rango intercuartílico (RIC) en variables no normales, para las variables cuantitativas y como frecuencia y porcentaje para las variables cualitativas. Los pacientes con y sin LRAPCY se compararon utilizando la prueba no paramétrica U de Mann-Whitney para muestras independientes. Las medidas repetidas de los biomarcadores (al inicio, a las 4 horas y a las 12 horas tras la administración del contraste) en la población total se compararon mediante la prueba no paramétrica de rangos con signo de Wilcoxon para muestras relacionadas. Todos los valores de p correspondieron a contrastes bilaterales, y aquellos <0,05 se consideraron estadisticamente significativos. Los análisis se realizaron con el programa IBM SPSS Statistics, versión 29.0.2.0 (North Castle Drive, MD-NC119. Armonk, NY 10504-1785, EEUU).

## RESULTADOS

Durante el periodo de estudio de cinco años, se evaluó la elegibilidad de 3.750 pacientes, de los cuales se excluyeron 3.672 (97,9%): 3.224 por alta precoz, 287 por negativa a participar y 161 por no cumplir con los criterios de alto riesgo. A pesar de que en nuestro hospital se realizan aproximadamente 3.500 TC con contraste intravenoso al año, muchos pacientes permanecen menos de 48 horas hospitalizados, lo que los hacía no elegibles para este estudio. Finalmente, se incluyeron 78 pacientes, pero solo se analizaron 62 debido a que nueve (11,5%) optaron por abandonar el estudio, seis (7,7%) tuvieron muestras incompletas y uno (1,3%) no recibió contraste por niveles elevados de creatinina basal.

Los datos demográficos y comorbilidades de los 62 pacientes de alto riesgo que recibieron iohexol intravenoso se presentan en la Tabla 1. La mayoría estaban ingresados en servicios médicos (n = 52; 84% frente a servicios quirúrgicos n = 10; 16%) y se sometieron a una TC abdominal o toracoabdominal (n = 50; 81%). Veinte pacientes (32,2%) tenían disfunción renal previa con FGe_(MDRD4)_ <60 mL/min/1,73 m^2^ (65% ERC G3A, 30% G3B y 5% G4), la mitad tenían hipertensión y todos ellos tenían DM. Además, 22 pacientes (35,5%) tenían al menos dos factores de riesgo para LRAPCY. Antes de la administración del contraste, la inmensa mayoría (92%) recibió hidratación y más de la mitad (58%) recibió N-acetilcisteína (NAC) intravenosa (n=31), oral (n=4) o ambas (n=1).

**Tabla 1:** Características demográficas globales y por lesión renal aguda postcontraste yodado

Variable	Global n=62	No-LRA n=43	LRAPCY n=19	p (χ2)
Edad (años)*	67,8 (14)	64,9 (14,9)	74,4 (9,1)	0,01*
Sexo (varón)	39 (63%)	29 (67%)	10 (53%)	0,2
IMC (kg/m^2^)*	26,7 (5,6)	26,9 (5,4)	26,0 (6,2)	0,57*
PAS (mm Hg)*	126 (20)	125 (21)	129 (20)	0,42*
PAM (mm Hg)*	90 (10)	89 (11)	90 (8)	0,79*
PAD (mm Hg)*	71 (8)	72 (9)	70 (8)	0,61*
*Comorbilidades*				
DM	39 (63%)	27 (63%)	12 (63%)	0,98
HTA	31 (50%)	18 (42%)	13 (68%)	0,05
ERC (FGe_(MDRD4)_ <60)	20 (32%)	8 (19%)	12 (63%)	<0,01
G3A	13 (21%)	6 (14%)	7 (37%)	0,004
G3B	6 (9,7%)	1 (2,3%)	5 (26,3%)	
G4	1 (1,6%)	1 (2,3%)	0	
AINES	29 (47%)	23 (53%)	6 (32%)	0,11
IECA	20 (32%)	12 (28%)	8 (42%)	0,27
Deshidratación en ayunas	15 (24%)	12 (8%)	3 (16%)	0,36
Anemia	54 (87%)	36 (84%)	18 (95%)	0,42
*Contraste*				
Fluidoterapia previa	57 (92%)	41 (95%)	16 (84%)	0,14
NAC iv	31 (50%)	18 (42%)	13 (68%)	0,05
Volumen de contraste (mL)	125 (15)	124 (14)	128 (16)	0,29*
MACD (mL)	407 (145)	432 (135)	352 (155)	0,03*
Vol/MACD (%)	35,4 (14,8)	32,1 (12,3)	42,9 (17,4)	0,02*

No-LRA: sin lesión renal aguda; LRAPCY: lesión renal aguda postcontraste yodado; *: media (desviación estándar) comparadas con U de Mann-Whitney; IMC: índice de masa corporal; PAS: presión arterial sistólica; PAM: presión arterial media; PAD: presión arterial diastólica; DM: diabetes; HTA: hipertensión arterial; ERC: enfermedad renal crónica por FGe_(MDRD)_ <60 mL/min/1,73 m^2^, categorizada en estadios KDIGO G3A, G3B y G4); AINES: uso de antiinflamatorios no-esteroides; IECA: uso de inhibidores de la enzima de conversión de la angiotensina o de antagonistas de los receptores de la angiotensina II; Anemia: hemoglobina <14 g/dL en varones y <12 g/dL en mujeres; NAC iv: N-acetilcisteina intravenosa previa al uso de iohexol; MACD: dosis de contraste máxima aceptable; Vol/MACD: mL de iohexol administrado/MACD.

Diecinueve pacientes (30,6%) desarrollaron LRAPCY: nueve con aumento en la creatinina sérica sin oliguria, nueve con oliguria sin aumento en la creatinina sérica >0,3 mg/dL, y uno con oliguria y aumento de creatinina. Los factores asociados con LRAPCY fueron una mayor edad, hipertensión arterial, ERC (12/20 = 60% en pacientes con ERC vs 7/42 = 16,7% sin ERC), y mayor volumen de contraste normalizado por la MACD. La administración de NAC previa al uso de iohexol no se relacionó con la incidencia de LRAPCY. La incidencia de LRAPCY se triplicó en pacientes con DM y ERC (12/20=60% frente a 4/19=21% con DM sin ERC). Ningún paciente requirió técnicas de depuración extrarrenal.

**Tabla 2:** Biomarcadores de función renal globales y por lesión renal aguda postcontraste yodado

Datos renales	Global Media (DE)	No-LRA Media (DE)	LRAPCY Media (DE)	p*
*Creatinina (mg/dL)*				
Basal	1,0 (0,38)	1,0 (0,4)	1,1 (0,4)	0,05
24 h	1,0 (0,38)	1,0 (0,3)	1,2 (0,4)	0,03
48 h	1,1 (0,6)^&^	0,9 (0,4)^&^	1,3 (0,8)^&^	0,03
*Cistatina (mg/L)*				
Basal	1,0 (0,34)	0,9 (0,2)	1,2 (0,5)	0,13
24 h	1,0 (37)	0,9 (0,3)	1,2 (0,5)	0,03
MDRD				
Basal	73,3 (26,4)	78,2 (23,8	62,0 (29,2)	0,02
24 h	70,0 (24,4)	76,0 (23,4)	56,7 (21,7)	0,004
48 h	74,8 (32,4)	84,7 (28,9)	57,8 (31,6)	0,005
*CKD-EPI creat-cist*				
Basal	78,6 (26,3)	84,3 (23,3)	65,7 (28,6)	0,02
24 h	77,5 (26,7)	83,9 (24,3)	62,9 (26,8)	0,007
48 h	74,7 (30,0)	84,3 (25,6)	58,7 (30,4)	0,005

No-LRA: sin lesión renal aguda; LRAPCY: lesión renal aguda postcontraste yodado; *: media (desviación estándar) comparadas con U de Mann-Whitney; &: estadísticos calculados sobre n=48 (global), n=30 (No-LRA) y n=18 (LRAPCY); MDRD: tasa de filtración glomerular estimada mediante MDRD-4; CKD-EPI creat-cist: tasa de filtración glomerular estimada mediante *Chronic Kidney Disease Epidemiology Collaboration* creatinina-cistatina.

Los datos de función renal medida y estimada se presentan en la Tabla 2. Quince pacientes (24%) tenían niveles elevados de cistatina antes de la administración del contraste. Los pacientes que desarrollaron LRAPCY tenían un filtrado glomerular basal estimado un 30% menor que aquellos que no desarrollaron disfunción renal.

Respecto a los biomarcadores renales basales y postcontraste (Tabla 3), solo el NGAL mostró una distribución normal, y no se encontraron diferencias significativas entre los pacientes que desarrollaron LRAPCY y los que no. La medición basal de IPF2 en orina mostró que 36 pacientes (58%) tenían niveles elevados antes de la administración del contraste. Los pacientes con nivel basal de NGAL en sangre elevado no tuvieron un desarrollo significativamente mayor de LRAPCY (7/20 = 35% frente a 12/42 = 29%; p = 0,6). Solo cuatro pacientes (6%) tuvieron un NephroCheck® Test elevado a las 12 horas de la administración del contraste, de los cuales solo uno desarrolló oliguria sin aumento de creatinina.

**Tabla 3:** Biomarcadores renales no-funcionales globales y por lesión renal aguda postcontraste yodado

Biomarcadores		Global	No-LRA	LRAPCY	p
		n = 62	n = 43	n = 19	(U-MW)
*Valores cuantitativos, mediana (RIC)*					
*NGAL, ng/mL*					
S	pre	269 (311)	258 (300)	327 (424)	0,39*
	4 h	204 (270)	198 (267)	217 (294)	0,81*
O	pre	20 (43)	21 (55)	16 (31)	0,34
	4 h	15 (33)	17 (50)	13 (31)	0,35
*NGAL/creat, ng/mg*					
O	pre	34 (85)	36 (138)	27 (86)	0,35
	4h	36 (152)	39 (155)	21 (123)	0,36
*IL-8, pg/mL*					
S	pre	27 (41)	24 (30)	28 (63)	0,46
	12h	31 (90)	31 (88)	31 (93)	0,8
O	pre	49 (99)	31 (26)	25 (35)	0,61
	12 h	21 (44)	19 (43)	26 (60)	0,71
*IL-8/creat, pg/mg*					
O	pre	49 (99)	46 (82)	51 (116)	0,93
	12 h	51 (126)	45 (139)	59 (100)	0,93
*CT-1, fmol/ml*					
S	pre	12 (13)	13 (13)	7 (10)	0,09
	4h	9 (12)	12 (11)	6 (13)	0,05
	12h	10 (13)	10 (15)	11 (11)	0,8
O	pre	2,2 (2,6)	2,4 (2,6)	1,5 (3,2)	0,24
	4h	1,3 (1,8)	1,4 (2,5)	1,2 (1,6)	0,39
	12h	1,3 (2,7)	1,2 (2,6)	1,5 (5,3)	0,42
*CT-1/creat, fmol/mg*					
O	pre	4,3 (5,0)	4,9 (6,0)	3,4 (5,5)	0,15
	4h	4,3 (8,1)	4,5 (9,8)	3,2 (5,5)	0,35
	12h	3,9 (9,0)	4,0 (5,4)	3,5 (12,7)	0,94
*SOD, U/mL*					
S	pre	5,5 (1,1)	5,3 (1,2)	5,6 (1,2)	0,58
	4 h	5,3 (1,3)	5,2 (1,3)	5,4 (1,5)	0,75
*IPF2, pg/mL*					
O	pre	3.632 (10.898)	3.517 (7593)	6.025 (19.088)	0,28
	12h	3.936 (17.646)	3.696 (5297)	8.237 (26.871)	0,06
*IPF2/creat, pg/mg*					
O	pre	7.149 (20.288)	6.421 (15.648)	9.977 (41.149)	0,27
	12h	12.358 (42.254)	1.006 (16.915)	20.389 (82.875)	0,1
*Nephrocheck (ng/mL)2/1.000*					
O	12 h	0,12 (0,11)	0,11 (0,09)	0,12 (0,14)	0,65
*Frecuencia de valores elevados, n (%)*					*χ2*
*NGAL*					
S >400 ng/mL	pre	20 (32)	13 (30)	7 (37)	0,43
	4 h	14 (23)	10 (23)	4 (21)	0,56
O>150 ng/mL	pre	4 (6)	4 (9)	0	0,22
	4 h	4 (6)	4 (9)	0	0,22
*IL-8*					
S>65 pg/mL	pre	12 (19)	7 (16)	5 (26)	0,27
	12h	21 (34)	14 (33)	7 (37)	0,48
O>200 pg/mL	pre	3 (5)	1 (2)	2 (11)	0,22
	12 h	3 (5)	1 (2)	2 (11)	0,22
*CT-1*					
S>40 fmol/mL	pre	2 (3)	1 (2)	1 (5)	0,52
	4h	2 (3)	1 (2)	1 (5)	0,52
	12h	1 (2)	0	1 (5)	0,27
*IPF2*					
O>3.000 pg/mL	pre	36 (58)	24 (56)	12 (63)	0,4
	12h	38 (61)	25 (58)	13 (68)	0,36

Mediana (rango intercuartílico) comparadas mediante U de Mann-Whitney (U-MW) excepto * con t de Student para muestras independientes; No-LRA: sin lesión renal aguda; LRAPCY: lesión renal aguda postcontraste yodado; S: determinación en sangre; O = determinación en orina; /creat : valor ajustado a la creatinina en orina; NGAL: lipocalina asociada a la gelatinasa de neutrófilos; IL-8:interleucina-8; CT-1: cardiotrofina-1; SOD: superóxido dismutasa; IPF2 : F2-isoprostanos urinarios.

**Tabla 4:** Evolución de los biomarcadores renales* tras la administración del contraste

	Precontraste	4 h	12 h	p 4h/pre	p 12h/pre
*Biomarcadores no-funcionales*					
*NGAL, ng/mL*					
S	269 (311)	204 (270)	-	<0,01	-
O	20 (43)	15 (33)	-	0,08	
*NGAL/creat, ng/mg*					
O	34 (85)	36 (152)	-	0,83	-
*IL-8, pg/mL*					
S	27 (41)	-	31 (90)	-	0,006
O	26 (26)	-	21 (44)	-	0,95
*IL-8/creat, pg/mg*					
O	49 (99)	-	51 (126)	-	0,32
*CT-1, fmol/mL*					
S	12 (13)	9 (12)	10 (13)	0,07	0,07
O	2,2 (2,6)	1,3 (1,8)	1,3 (2,7)	0,09	0,21
*CT-1/creat, fmol/mg*					
O	4,3 (5,0)	4,3 (8,1)	3,9 (9,0)	0,81	0,92
*SOD, U/mL*					
S	5,5 (1,1)	5,3 (1,3)	-	0,046	-
*IPF2, pg/mL*					
O	3.632 (10.898)	-	3.936 (17.646)	-	0,17
*IPF2/creat, pg/mg*					
O	7.149 (20.218)	-	12.358 (42.254)	-	0,08
*Biomarcadores funcionales*					
Creatinina	1,0 (0,38)	1,0 (0,38)	1,1 (0,6)^&^	0,37	0,18
Cistatina	1,0 (0,34)	1,0 (0,37)	-	0,98	-
MDRD	73,3 (26,4)	70,0 (24,4)	74,8 (32,4)	0,67	0,79
CKD-EPI creat-cist	78,6 (26,3)	77,5 (26,7)	74,7 (30,09)	0,61	0,33

*: Mediana (rango intercuartílico) comparadas mediante la prueba de rangos con signo de Wilcoxon para muestras relacionadas; S: determinación en sangre; O: determinación en orina; /creat: valor ajustado a la creatinina en orina; NGAL: lipocalina asociada a la gelatinasa de neutrófilos; IL-8: interleucina-8; CT-1 : cardiotrofina-1; SOD: superóxido dismutasa; IPF2: F2-isoprostanos urinarios; &: estadísticos calculados sobre n = 48 (a las 12 h). CKD-EPI creat-cist: tasa de filtración glomerular estimada mediante *Chronic Kidney Disease Epidemiology Collaboration* creatinina-cistatina.

También se estudió la evolución de los biomarcadores tras la administración del contraste (Tabla 4). Observamos una disminución en los valores de SOD y NGAL en sangre a las 4 horas, junto con un aumento de IL-8 en sangre a las 12 horas de la administración del contraste, sin cambios significativos en el resto de los biomarcadores.

## DISCUSIÓN

En nuestra muestra prospectiva de pacientes con alto riesgo de daño renal que recibieron contraste yodado intravenoso, el 30,6% desarrollaron lesión renal aguda. Este resultado se asoció con mayor edad, disfunción renal previa y un mayor volumen de contraste normalizado por la MACD, sin diferencias en los biomarcadores no funcionales. Observamos que la administración de contraste yodado se relacionó con una disminución de la actividad antioxidante de la SOD a las 4 horas y un aumento de IL-8 a las 12 horas.

Nuestros hallazgos respaldan la nefrotoxicidad potencial de los contrastes yodados. A pesar del uso de contraste no iónico y de baja osmolaridad, administrado por vía intravenosa en lugar de intraarterial, y la implementación de medidas preventivas como la hidratación y la administración de NAC, no pudimos prevenir la LRAPCY en casi un tercio de los pacientes.

El principal factor de riesgo para la LRAPCY es la ERC preexistente. Hasta la fecha, ningún estudio controlado^13^ ha identificado un riesgo de LRAPCY en pacientes con una FGe ≥45 mL/min/1,73 m^2^. Sin embargo, en nuestro estudio, 53 pacientes (85%) tenían FGe ≥45 y doce de ellos (23%) sufrieron LRAPCY, lo que indica el alto riesgo de nuestra población y la dificultad para distinguir entre el daño renal producido por el contraste yodado o asociado a su uso en la evolución de pacientes con riesgo renal^1^.

El volumen de contraste administrado, ajustado a la MACD, fue uno de los factores asociados con una mayor incidencia de LRAPCY. La reducción del volumen de contraste yodado administrado, junto con la hidratación previa, son las dos principales medidas preventivas recomendadas^14^. La administración de NAC como prevención de LRAPCY presenta resultados discordantes en la literatura^15^. Sin embargo, en la práctica se utiliza con frecuencia debido a su seguridad, bajo coste y facilidad de aplicación, además de los resultados favorables informados en algunos metaanálisis^14^.

En la valoración de los biomarcadores destaca la gran variabilidad y dispersión de nuestros resultados, consecuencia en parte de la heterogeneidad de nuestra población. A pesar de ello, hemos detectado una disminución de la actividad antioxidante a las 4 horas y una elevación de la IL-8 a las 12 horas tras la administración del contraste. Estos resultados son compatibles con un aumento inflamatorio o del estrés oxidativo producido por el contraste yodado.

La SOD es el sistema antioxidante natural que previene la citotoxicidad y el daño tisular mediados por ROS. El aumento en la generación de ROS y la disminución de la SOD después de la exposición a contrastes yodados se ha observado en varios estudios *in vitro* e *in vivo*^5^. El estrés oxidativo también se evidencia por un IPF2 basal elevado en orina en un 58% de nuestros pacientes, secundario a su patología de base oncológica o infecciosa, cuyos niveles aumentan tras la administración de iohexol, aunque no alcanzó significación estadística.

El incremento observado de IL-8 a las 12 horas tras la administración del iohexol puede reflejar la inflamación provocada por este contraste yodado. La IL-8 es una citocina de naturaleza proinflamatoria sintetizada en fibroblastos, células endoteliales, monocitos, macrófagos y células dendríticas. Su aumento se ha descrito en daño renal asociado a cirugía cardiaca y a sepsis^16^, produciendo quimiotaxis de neutrófilos, formación de lípidos bioactivos, amplificación de la inflamación local y estimulación de la angiogénesis. La respuesta proinflamatoria reflejada por el aumento de IL-8 no se ha visto acompañada por un incremento de otros biomarcadores inflamatorios como NGAL o CT-1.

La NGAL en plasma u orina representa un biomarcador predictivo temprano con muy buen desempeño en la predicción de la LRAPCY^17^. En nuestra cohorte observamos disminución de NGAL a las 4 horas postcontraste, mientras que Filiopoulos y col^18^ describieron una elevación significativa de NGAL en plasma 6 horas tras la administración de contraste intravenoso entre pacientes hospitalizados sometidos a TC electiva con contraste. Esta discrepancia podría deberse a la hidratación previa que recibieron la mayoría de nuestros pacientes.

La cardiotrofina-1 (CT-1) es un miembro de la familia IL-6 de citoquinas con potente efecto antiapoptótico sobre hepatocitos, cardiomiocitos y neuronas, protegiéndolos frente a daños tóxicos o isquémicos^19^; en estudios animales también ha demostrado protección contra el daño funcional y estructural renal provocado por contrastes yodados^20^. En nuestros pacientes no hemos observado modificaciones significativas en los niveles plasmáticos o urinarios de esta citoquina.

Nephrocheck® es un biomarcador con buen rendimiento diagnóstico y una herramienta predictiva para la detección de LRA en pacientes sometidos a cirugía mayor o a cirugía cardíaca, con inestabilidad hemodinámica o con sepsis^21^. Mide dos proteínas en la orina, TIMP-2 y IGFBP-7, que se expresan y secretan en el riñón causando la detención del ciclo celular en la fase G1 durante una fase muy temprana del daño celular, en respuesta a diversas agresiones (por ejemplo, estrés oxidativo, toxinas, isquemia, sepsis, inflamación). En nuestro trabajo, al igual que otros autores^22^, no hemos hallado incrementos significativos del Nephrocheck®, lo cual podría explicarse por ser una prueba cuyo desarrollo y validación han sido en situaciones clínicas donde el potencial daño renal es previsiblemente mayor (cirugía cardiaca o sepsis).

Pensamos, de acuerdo con otros autores^23^, que la TC con contraste no debe posponerse si es necesaria para el diagnóstico de una afección potencialmente mortal, ya que el riesgo incierto de LRAPCY, dada la ausencia de cambios relevantes en los biomarcadores de lesión renal, debe sopesarse con el riesgo de pasar por alto un diagnóstico importante. Aunque siempre debemos tener en cuenta la posibilidad de utilizar procedimientos de imagen que no requieran contrastes yodados, en los pacientes de riesgo utilizaremos las medidas profilácticas que han demostrado eficacia: una adecuada hidratación previa y posterior al contraste, la interrupción de otros nefrotóxicos (AINES, diuréticos de asa, aminoglucósidos, metformina...), el uso de contraste basado en dímeros no iónicos con osmolaridad similar a la plasmática (iodixanol^24^), y en la menor dosis posible.

Las limitaciones de nuestro estudio son las siguientes: El tamaño muestral es limitado, con tan solo 62 pacientes, lo que reduce la potencia para detectar diferencias significativas entre los pacientes con y sin daño renal, produce estimaciones menos precisas de los efectos del contraste yodado y limita la generalización de los hallazgos, haciéndolos menos fiables y más susceptibles a la influencia de valores atípicos o variabilidad aleatoria. Se trata de un estudio sin grupo control (pacientes que no hubieran recibido contraste yodado), lo que nos impide diferenciar el daño producido por el contraste yodado del asociado a otras patologías presentes en los pacientes. Hay variables clínicamente importantes que no están recogidas, como el sedimento urinario, variables sistémicas de inflamación (proteína C reactiva, procalcitonina, etc.) o el diagnóstico clínico de indicación de la TC. La omisión de estas variables introduce la posibilidad de factores de confusión no controlados que pueden influir en la relación observada entre el contraste yodado y el fallo renal, compromete la capacidad de hacer inferencias causales y limita la interpretación de los resultados, ya que no podemos determinar si las variables no recogidas podrían haber alterado significativamente los hallazgos.

No obstante, el tamaño muestral es parecido al de estudios similares previos de biomarcadores, y nuestra muestra destaca por ser prospectiva, con pacientes de alto riesgo renal, no ambulatorios, y con un riguroso análisis y seguimiento de biomarcadores, algunos novedosos y no descritos previamente, como la CT-1 urinaria.

En conclusión, nuestro estudio muestra una alta incidencia de LRAPCY en pacientes hospitalizados de alto riesgo, subrayando la importancia de una evaluación cuidadosa de los factores de riesgo antes de la administración de contraste yodado. La edad avanzada, la disfunción renal previa y un mayor volumen de contraste administrado son factores asociados con un mayor riesgo de LRAPCY. Aunque los biomarcadores de inflamación y estrés oxidativo mostraron cambios post-contraste, no se correlacionaron significativamente con la incidencia de LRAPCY. Estos hallazgos sugieren que, además de los factores clínicos tradicionales, se necesitan nuevas estrategias y biomarcadores más específicos para predecir y prevenir la LRAPCY.

Futuras investigaciones deberían centrarse en identificar mecanismos moleculares y celulares adicionales implicados en la patogénesis de LRAPCY, así como en desarrollar intervenciones efectivas para reducir su incidencia en poblaciones vulnerables.
